# Residual dorsal displacement following surgery in distal radial fractures: A cause for trouble?

**DOI:** 10.1007/s00068-022-02061-3

**Published:** 2022-07-26

**Authors:** Steffi S. I. Falk, Thomas Mittlmeier, Georg Gradl

**Affiliations:** 1 Clinic of Trauma, Hand and Reconstructive Surgery, University of Rostock, Schillingallee 35, 18055 Rostock, Germany; 2 Munich Municipal Hospital Group, Clinic of Trauma, Orthopaedic, Hand and Reconstructive Surgery, Clinic Harlaching, Sanatoriumsplatz 2, 81545 Munich, Germany

**Keywords:** Distal radius fracture, Volar tilt, Osteosynthesis, Surgical technique, Osteoporotic fracture

## Abstract

**Purpose:**

Distal radius fractures have great impact on activities of daily living of affected patients. Repeatedly, a non-anatomic restoration of the volar tilt can be observed in a minimum of 20% in postoperative X-ray control examinations. Hence, the question arises whether the achieved reduction is functionally acceptable, or whether a further attempt should be made to improve the surgical outcome.

**Methods:**

The data presented here originate from a prospective analysis including three therapy studies on surgical treatment options for fractures of the distal radius between 2004 and 2011. For this study, the participants were divided into two groups: The first group represents the cases with non-anatomical restoration of the volar tilt with − 5° to 5°. The second group contains patients with an anatomical volar tilt between 6° and 15°.

**Results:**

A total of 624 patients were screened according to the inclusion criteria. Radiological evaluation showed consolidation of all fractures. The mean volar tilt as measured in standard x-rays of the wrist was 0° and 8°, respectively. The range of wrist motion in relation of the healthy opposite side was comparable in all directions (for example comparison group 1: Ext/Flex 94/94%; group 2: Ext/Flex 93/93%). Functional assessment of postoperative midterm results employing the Castaing and Gartland & Werley scores 2.3 years after surgery did not reveal significant differences between both groups.

**Conclusion:**

According to the available data, a volar tilt in the range of − 5° to 5° can be tolerated intraoperatively without any risk of loss of function regarding the patient's manual abilities.

## Introduction

Fractures in older people have become a major health problem in most countries. Among these, distal radius fractures are the most common [1–4] and their incidence is increasing [5, 6]. Outcome assessment is becoming more and more critical in evaluating the effectiveness of surgical interventions [7]. Such assessments can help distinguish between different treatment options and identify effective options, which in turn can enhance patient care.

Good wrist function is synonymous with the ability to care independently for oneself in everyday life [8]. Thus, the functional outcome after distal radius fracture is essential for the quality of life and the prevention of long-term disability [9, 10].

The basic treatment options are the non-operative one and various surgical therapies. Non-operative therapy generally involves immobilisation in a plaster cast for 6 weeks. Surgical therapies include, for example, open reduction and internal fixation by means of a palmar and/or dorsal plate, a plate combination, an interlocking nail, the wrist bridging and non-bridging external fixator and k-wire transfixation in combination with cast immobilisation.

There is still no broad consensus on the gold standard in the treatment of distal radius fractures [11, 12]. Surgical therapy is now established for the unstable distal radius extension fracture. Fractures with the following criteria are considered as unstable distal radius fractures**:**

- initial radial shortening of more than 5 mm,
- fragmentation of at least 50% of the volar-dorsal distal radius,
- initial fragment dislocation of more than 1 cm,
- intra-articular multi-fragmentary fracture,
- concomitant distal ulna fracture,
- pronounced osteoporosis,
- initial volar tilt of more than − 20°,
- metaphyseal fracture comminution zone [13].

Regardless of the implant chosen (plate variants, k-wires, external fixator, intramedullary nail), the common goal is to restore a good wrist function. The intraoperative benchmark used for a good wrist function is restoration of the anatomy [14–16]. Consequently, it is recommended to achieve a continuous congruent joint surface, an ulnar variance of 0 to − 2 mm and a radioulnar inclination of about 24° as well as a volar tilt of 10° [17–19].

So far, it is unclear whether the requirement for restoration of volar tilt of the meta/epiphyseal fragment is mandatory for a favourable outcome, and this question is controversial in the literature [20, 21]. According to Dario et al. [11] and Cai et al. [22], the restoration of volar tilt is, along with ulnar variance, the most crucial anatomical feature for good function. The literature gives very different results for the achieved postoperatively more than anatomical volar tilt. The percentage of patients in whom an anatomical volar tilt is achieved postoperative ranges from 0 over 4 up to 80 percent [23–25]. The surgeon is then faced with the question whether the achieved reduction is acceptable, or whether a further attempt should be made to improve it. Of course, the risk of increasing instability in the fracture due to enlargement of the existing fracture zone and the associated loss of fracture reference must not be neglected.

With the advances in medicine, our patients' expectations of the treatment outcome are also increasing. Whereas in the past freedom from pain was sufficient, nowadays patients expect complete restoration of function [26]. However, it is not always possible to achieve the normal anatomy. Consequently, the question arises to what degree the original anatomy is to be restored in order to achieve an acceptable function with regard to a patient's high demands.

Since we excluded patients with a loss of length of the distal radius of 2 mm, we present here, to our knowledge, the first study that examines only the influence of a volar tilt of up to − 5° on the functionality and pain at the wrist after up to 2 years following surgical fracture treatment.

## Materials and methods

The data presented here originate from a retrospective analysis of patient data from previously completed studies on the surgical treatment of distal radius fractures. Between January 2004 and August 2011, patients with unstable distal extension radius fractures (AO type A3.2, A3.3 and C fractures) were prospectively included in three randomized studies on treatment alternatives. The procedures used were palmar plate osteosynthesis (2.4 mm, Synthes, USA and 2.4 mm M.O.R.E. Medical Solutions, Rostock, Germany) following open reduction, intramedullary interlocking nailing (Targon DR, Aesculap-BBraun, Tuttlingen, Germany (c)) and a non-bridging external fixator (DePuy Synthes, Johnson&Johnson Medtech, Freiburg, Germany) after closed fracture reduction.

The studies considered compared the surgical management of (I) open reduction and palmar plate versus intramedullary interlocking nail [15], (II) open reduction and palmar plate versus non-bridging external fixator [18] and (III) palmar plate with the placement of one versus two distal rows of palmar plate (unpublished data).

Patients were followed up at 8 weeks and 12 months after surgery, respectively. Since in two of the newly evaluated studies the patients were re-examined after 2 years, these results were also taken into account. The two last mentioned studies also include all three implants considered here.

The follow-up examinations after 8 weeks, 12 and 24 months were mainly carried out clinically and radiologically, but only clinically or by telephone at the patient's request. At all times in the clinical and radiological follow-up examinations, the range of wrist joint motion was measured using a goniometer. In addition, hand strength was determined via Jamar dynamometry and a conventional x-ray of the operated wrist was taken in anterior–posterior and lateral projection. Radial shortening was assessed by measuring the distance between the ulnar border of the distal radius and the distal articular surface of the ulna. The evaluation of the radiological images of all three studies was performed by one physician in advanced training and supervised by one experienced consultant trauma surgeon. In the radiological images, both volar tilt and radial shortening were determined. In addition, the x-rays were examined for osseous consolidation and eventual implant failure. Furthermore, the scores according to Castaing [27] and Gartland & Werley [28] were determined as well as the existing pain conditions using the numerical visual analog scale.

In order to minimise possible biases due to age or different life stresses (like wrist-straining hobbies, sports activities or manual labour) of the wrists of the study participants, the assessment of the range of motion was always compared with the uninjured opposite side. Therefore, the data for the range of motion are given as percentages which could reach a maximum of 100. Consequently, patients with previous wrist joint injuries at the opposite side were excluded. Patients with systemic joint diseases and possible impairment of joint function, e.g. rheumatoid arthritis, were also excluded.

Within this evaluation, only data with complete clinical/radiological follow-up and a volar tilt ranging from − 5° to 15° were selected. A negative value for volar tilt here represents a remaining dorsal tilt while positive numerical values represent a volar tilt.

For the evaluation of functionality in relation to volar tilt, the study participants were divided into two groups. The first group represents the non-anatomical restoration with − 5° to 5. The first group was compared with patients with an anatomical volar tilt between 6° and 15°.

A power analysis carried out in advance with G*Power resulted in a minimum number of participants of 86 per group for a power of 0.9 with an alpha of 0.05 and an effect size according to Cohend's of *d* = 0.5. For a power of 0.8, 64 patients per group would be sufficient.

The descriptive data were analysed using Microsoft Excel 2019. For further statistical analysis, the data were evaluated with SPSS version 27. After testing for normal distribution using the Kolmogorov-Smirnova test, the groups were examined using the Mann–Whitney *U* test as the data were not normally distributed. P-values below 0.05 or greater 0.95 were considered significant.

## Results

**Fig. 1:**
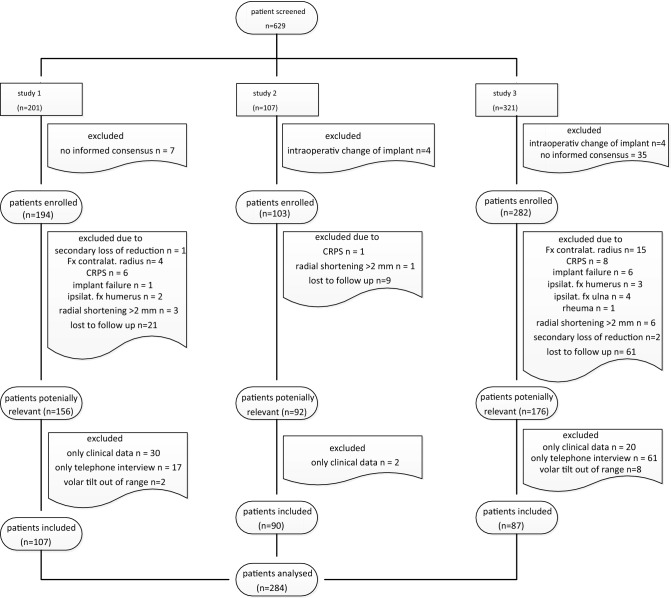
The patient population among the three studies considered. The inclusion criteria were met by 112 patients from the first study (*n* = 201; 56%), 90 patients from the second study (*n* = 107; 84%) and 87 patients from the third study (*n* = 307; 28%)

A total of 624 patients from three studies were screened according to the inclusion criteria. Complete data at the 12 months follow-up could be identified in a total of 284 study participants (Fig. 1).

**Fig. 2:**
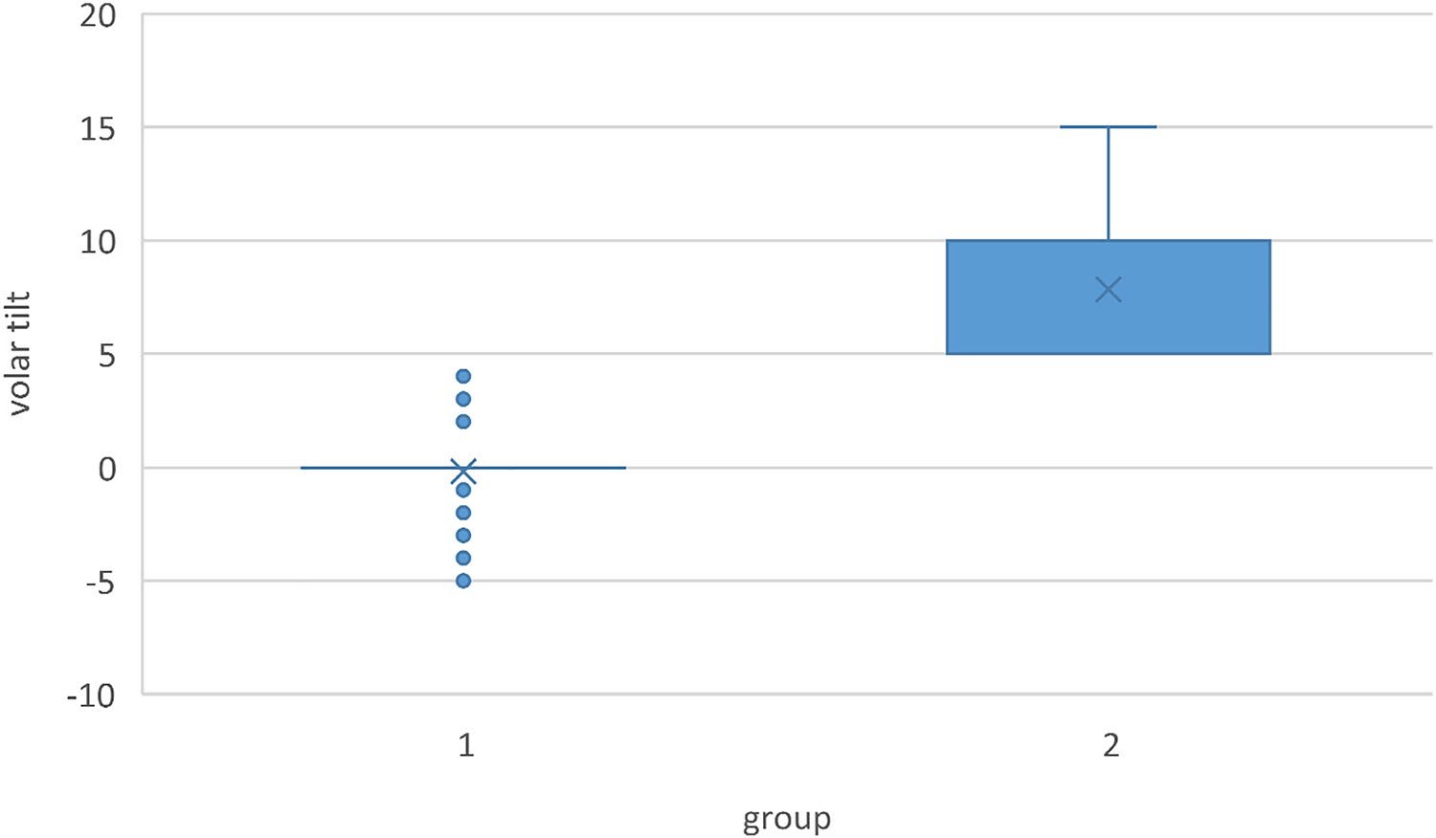
Box plot diagram of the volar tilt of both patient groups studied. Group 1 represents the non-anatomical restoration with − 5° to 5° and group 2 patients with an anatomical volar tilt between 6° and 15°

Of these 284 patients, 169 were in the group with a volar tilt of less than 5° (group 1). Group two with study participants with a volar tilt of at least 5° included 115 patients. With 63 and 64 years, respectively the mean age was almost identical in both groups. The mean volar tilt was − 0.2° and 7.9°, respectively (Fig. 2). The breakdown of group characteristics is shown in Table 1.

**Table 1:** List of descriptive characteristics of the study for the group of patients with volar tilt less than 5° (group 1) and greater than or equal to 5° (group 2)

*n* = 284	Group 1 (*n* = 169)	group 2 (*n* = 115)
Age	64 (13)	63 (17)
Gender		
Male	20	15
Female	149	100
Surgical technique		
Palmar plate	126	65
Nail	36	15
External fixator	7	35
AO fracture classification		
Type A	121	84
Type C	48	31

The age is given with the standard deviation in brackets

The 1-year follow-up data were examined in both groups at an average of 13 months after surgery (range 9–19 months).

The range of motion given in percent of the intact opposite side was comparable in all planes except for flexion (group 1: extension/flexion 91/84%; group 2: extension/flexion 92/90%). A detailed list as well as the Castaing and Gartland & Werley scores are given in Table 2. The radiological evaluation showed consolidation of all fractures after this period.

**Table 2:** Comparison of both groups after an average of 1 year after surgery

	Group 1	Group 2	*p*-value
Follow-up (month)	13.6 (1.7)	13.4 (1.8)	0.3251
Grip strength	80 (23)	85 (19)	0.0640
Range of motion			
Extension	91 (15)	92 (14)	0.4729
Flexion	84 (17)	90 (14)	0.0045
Pronation	99 (5)	100 (3)	0.2018
Supination	98 (7)	97 (9)	0.8913
Radial deviation	91 (16)	90 (17)	0.6502
Ulnar deviation	89 (16)	88 (16)	0.5591
Scores			
Castaing	2.4 (2.9)	1.8 (2.1)	0.1027
Gartland & Werley	2.4 (3.3)	2.0 (2.8)	0.3872
Pain			
VAS (rest)	0.3 (1.2)	0.2 (1)	0.5978
VAS (motion)	1.6 (4.0)	1.1 (1.9)	0.3489
Radiographic evaluation			
Volar tilt (°)	− 0.2 (1.9)	7.9 (3.8)	0.0001
Radial shortening (mm)	0.1 (0.8)	0.3 (0.7)	0.0164

The range of motion as well as the grip strength are given as a percentage of the uninjured opposite side with the standard deviation in brackets. The follow-up time given in the table is in months

The 2-year follow-up examination took place after an average of 2.3 years after 28 months. At this follow-up, 82 patients could be examined in each patient group. The significantly lower number of patients results from the fact that only in two of the three studies there was a follow-up of the patients after 2 years. The range of motion in percent compared with the uninjured opposite side was comparable in all levels (group 1: extension/flexion 94/94%; group 2: extension/flexion 93/93%). A detailed list as well as the Castaing and Gartland & Werley scores is given in Table 3. Again, radiological evaluation showed consolidation of all fractures.

**Table 3:** The comparison of both groups after an average of 2.3 years after surgery

	Group 1	Group 2	*p*-value
Follow-up (month)	30.1 (6.5)	29.3 (5.5)	0.7516
Grip strength	87 (13)	93 (10)	0.0087
Range of motion			
Extension	94 (10)	93 (9)	0.5906
Flexion	94 (9)	93 (10)	0.6560
Pronation	99 (2)	99 (2)	0.4740
Supination	98 (6)	99 (4)	0.5642
Radial deviation	92 (13)	94 (8)	0.6509
Ulnar deviation	91 (13)	92 (11)	0.9515
Scores			
Castaing	1.7 (1.9)	1.6 (1.1)	0.8326
Gartland & Werley	2.1 (2.6)	1.8 (2.4)	0.4275
Pain			
VAS (rest)	0.1 (0.6)	0 (0.1)	0.2169
VAS (motion)	0.9 (1.8)	0.3 (0.9)	0.0772

The range of motion as well as the grip strength are given as a percentage of the uninjured opposite side with the standard deviation in brackets. The follow-up period is given in the table in months

## Discussion

The results presented here did not show any clinically relevant influence of the volar tilt following surgical treatment of distal radius fractures in the range of − 5° and 15° on wrist function or pain. In the follow-up examination 12 months after surgery, no significant difference for pain, hand strength and the clinical scores was detectable. The range of motion except for flexion was also equally good in both groups. After 2 years, the comparison of both groups yielded no significant differences for range of motion including flexion ability, pain and scores. The age and gender composition of the patient population studied corresponded with the one observed in epidemiological studies on distal radius fractures [2, 6, 29].

The first evaluated follow-up showed good results for all ranges of motion with a functional recovery of at least 84% of the opposite side. The range of motion achieved after 12 months was comparable with the one reported in other published studies [30–34]. Equally, the patients with the lower volar tilt showed excellent results, also with respect to the Castaing and Gartland & Werley scores.

Studies dealing with the effect of radial fractures that have not healed in an anatomical position on function are rare. Cai et al. [22] showed that radial height and volar tilt are the decisive prognostic anatomical factors, but did not give any information on the magnitude of the loss of function. Synn et al. [30] also addressed this issue and simultaneously examined 5 criteria. These included a volar tilt of − 10°. They found a greater loss of grip strength and a tendency towards a lesser range of motion in radial-ulnar-deviation. Likewise, Kong et al. [35] compared patients with non-acceptable anatomical restoration with patients with acceptable ones. Again, a volar tilt of -10° was one of the criteria. The patients with unacceptable restoration of anatomy showed a loss in grip strength, flexion and ulnar deviation. In the subsequent regression analysis, Kong et al. showed that flexion was particularly influenced by volar tilt. No information was given on the exact angle of volar tilt in the patient groups studied. The group around Dario et al. [11] also examined patients and saw a worse function with non-anatomical volar tilt (here outside 7°–15°) as well as an existing ulnar shortening (here > 1.5 mm). There was no information given on angles and the extent of functional impairment. However, Dario et al. also saw radial length and volar tilt as the crucial radiological parameters.

Since we excluded patients with a loss of length of the distal radius of 2 mm and more, we present here, to the best of our knowledge, the first research study that examines exclusively the role of the extent of volar tilt on wrist function.

Consistently with the expectation that palmar flexion would be lower with less volar tilt, it was significantly lower at six percent after 1 year. Given that six percentage points difference in an assumed range of motion of 60° flexion means 3.6°, the probability that this mathematically significant difference is also of clinical relevance is very low. This is also reflected in the consideration of flexion in the scores of Castain and Gartland & Werley [27, 28]. Both scores weight a functional limitation in flexion significantly less than in extension, ulnar deviation and also in supination. Regardless of clinical relevance, flexion ability recovered substantially in the following 12 months. Taking this development into account the data show that a lower volar tilt is not associated with a poorer range of motion in the long run.

The significantly lower hand strength after 2 years in the group with the lower volar tilt was unexpected. Both groups showed a significant increase in strength after 2 years compared with the 1-year follow-up. The group with the non-anatomical volar tilt recovered one percent more than the group with the anatomic tilt; without statistical significance. Ultimately, this resulted in an improvement of about seven percent in both groups. After 2 years, the grip strength in group two was six percent better than in group one. To what extent six percent are clinically important when the strength recovery is around 90 percent of the opposite side remains questionable. Studies on the minimum clinically important difference (MCID) found a difference of 6.5 kg, which is said to correspond to a difference of 19% [36, 37]. With the knowledge of the MCID, the specific grip strength was significantly decreased in the group with the lower volar tilt, but apparently not clinically relevant. Thus, the hand strength of both groups was comparable and corresponded to a very good functional result. Nevertheless, it would be interesting to see, if there was a further recovery of function beyond the observation period of our study.

The follow-up period of 2 years presented in this examination was significantly longer than in most other related publications. Usually, studies on the treatment of distal radius fractures are limited to an observation period of only up to 12 months [38–42]. Accordingly, our follow-up time was twice as long as in most other studies, and therefore represent the first midterm results for the question addressed here.

We'd like to point out that the size of both patient groups is much larger than any other group size published so far. Other studies found in the literature often showed group sizes of just around 30–40 patients. With 115 and 169 patients in our two groups, group sizes were almost three times larger than most studies. These numbers underline the robustness of the findings [39–41].

The methods used for the follow-up examination with measurement of hand strength and mobility correspond to the standard. Likewise, the scores used according to Caistaing and Gartland & Werley had widely been used [43–48]. Similarly, the assessment of existing pain using the VAS scale is accepted and known as a reliable measurement tool [44, 49, 50].

In contrast to other publications, in the current evaluation the range of motion was not given in degrees, but as a percentage of the mobility of the opposite side. This offers the advantage that comparisons across different age groups are possible. By definition, the injured side cannot be better than the healthy opposite side. A value of 100 percent represents a complete restoration of functionality in the direction of movement under consideration. Therefore, restrictions of movement due to age and wear and tear do not form a bias. In addition, the gender-specific difference is neutralized and the difference due to handedness is minimized [12, 51–53].

### Limitations

The data from our study were derived from three independent studies which had not been conducted specifically for the purpose analysed here. A particular study to scrutinize non-anatomical vs. anatomical reduction of the epiphyseal fragment of the distal radius might be problematic in terms of receiving a corresponding permit by the ethical committee. The same might apply, to convince the patient to participate in a study to prove that a somehow worse anatomical fracture position is not associated with poorer wrist function. Despite the chosen post-hoc analysis, it does represent an advantage that the original data from the three studies came from prospective randomized studies. Therefore, no major bias is to be expected in the patient clientele.

In addition, the number of patients, just below 300, is rather small to make a generally valid statement. However, compared to the published studies [39–41], the number of study participants in this evaluation is above average. With a total of 284 patients, we also achieved a good statistical power of over 0.9 for the study presented here.

The three treatment techniques used (angle-stable plate, external fixator and interlocking nail) are not represented in equal proportions in the groups. The patients remain comparable because none of the procedures involved joint fusion, even temporarily. Consequently, the operation procedure had no influence on postoperative range of motion exercises. In addition, patients of all procedures were able to exercise immediately postoperatively without movement limitation while maintaining a partial load bearing for 6 weeks. Thus, all study participants experienced the same postoperative rehabilitation procedure. The advantage of different implants included is that the results are not limited to one implant type.

Compared to the literature [30–34], the follow-up period of 2 years corresponds to midterm follow-up, only but, to the best of our knowledge there are currently no long-term evaluations available. On the other hand, the current and previously published therapy studies do not show longer periods, but rather shorter ones with an average of 12 months. Only with regard to a potential development of posttraumatic osteoarthritis, no final statements can be made due to the lack of long-term results. Here, a follow-up examination 10 years after surgery would certainly point the way again.

## Conclusion

The available data cannot support the requirement for an anatomical volar tilt of 10°. The results presented here allow the conclusion that very good wrist function can be achieved after 20 years, even taking into account that flexion and grip strength in the non-anatomical group are not optimal in the first year. According to the available data, a volar tilt of up to minus 5° can be tolerated intraoperatively without expecting any loss of wrist function.

With our data in mind, the decision whether to choose an operative or conservative treatment, should be reassessed. Conservative treatment may be a good option for a larger group of patients than previously—without having to reckon with movement restrictions and functional limitations.

## References

[1] Ateschrang A, Stuby F, Werdin F (2010). Flexor tendon irritations after locked plate fixation of the distal radius with the 3.5 mm T-plate: identification of risk factors. Z Orthop Unfall.

[2] Baruah RK, Islam M, Haque R (2015). Immobilisation of extra-articular distal radius fractures (Colles type) in dorsiflexion. The functional and anatomical outcome. J Clin Orthop Trauma..

[3] Bohannon RW (2019). Minimal clinically important difference for grip strength: a systematic review. J Phys Ther Sci.

[4] Brogren E, Petranek M, Atroshi I (2007). Incidence and characteristics of distal radius fractures in a southern Swedish region. BMC Musculoskelet Disord.

[5] Brogren E, Petranek M, Atroshi I (2015). Cast-treated distal radius fractures: a prospective cohort study of radiological outcomes and their association with impaired calcaneal bone mineral density. Arch Orthop Trauma Surg.

[6] Chung KC, Spilson SV (2001). The frequency and epidemiology of hand and forearm fractures in the United States. J Hand Surg Am.

[7] Billig JI, Sears ED, Travis BN (2020). Patient-reported outcomes: understanding surgical efficacy and quality from the patient’s perspective. Ann Surg Oncol.

[8] Court-Brown CM, Caesar B (2006). Epidemiology of adult fractures: A review. Injury.

[9] González N, Aguirre U, Orive M (2014). Health-related quality of life and functionality in elderly men and women before and after a fall-related wrist fracture. Int J Clin Pract.

[10] Vergara I, Vrotsou K, Orive M (2016). Wrist fractures and their impact in daily living functionality on elderly people: a prospective cohort study. BMC Geriatr.

[11] Dario P, Matteo G, Carolina C (2014). Is it really necessary to restore radial anatomic parameters after distal radius fractures?. Injury.

[12] Ochen Y, Peek J, van der Velde D (2020). Operative vs nonoperative treatment of distal radius fractures in adults: a systematic review and meta-analysis. JAMA Netw Open.

[13] Endres HG, Dasch B, Lungenhausen M (2006). Patients with femoral or distal forearm fracture in Germany: a prospective observational study on health care situation and outcome. BMC Public Health.

[14] Falk SS, Mittlmeier T, Gradl G (2016). Results of geriatric distal radius fractures treated by intramedullary fixation. Injury.

[15] Falk S (2016). Der Targon DR: Ein intramedullärer Kraftträger zur Behandlung der distalen Radiusfraktur.

[16] Geerts RW, Toonen HG, van Unen JM (2011). A new technique in the treatment of distal radius fractures: the Micronail®. Acta Orthop Traumatol Turc.

[17] Gologan RE, Koeck M, Suda AJ (2019). > 10-year outcome of dislocated radial fractures with concomitant intracarpal lesions as proven by MRI and CT. Arch Orthop Trauma Surg.

[18] Gradl G, Wendt M, Mittlmeier T (2013). Non-bridging external fixation employing multiplanar K-wires versus volar locked plating for dorsally displaced fractures of the distal radius. Arch Orthop Trauma Surg.

[19] Gradl G, Falk S, Mittlmeier T (2016). Fixation of intra-articular fractures of the distal radius using intramedullary nailing: a randomized trial versus palmar locking plates. Injury.

[20] Handoll HH, Madhok R (2009). WITHDRAWN: Surgical interventions for treating distal radial fractures in adults. Cochrane Database Syst Rev.

[21] Jerrhag D, Englund M, Karlsson MK (2017). Epidemiology and time trends of distal forearm fractures in adults—a study of 11.2 million person-years in Sweden. BMC Musculoskelet Disord.

[22] Cai L, Zhu S, Du S (2015). The relationship between radiographic parameters and clinical outcome of distal radius fractures in elderly patients. Orthop Traumatol Surg Res.

[23] Madsen ML, Wæver D, Borris LC (2018). Volar plating of distal radius fractures does not restore the anatomy. Dan Med J.

[24] Mignemi ME, Byram IR, Wolfe CC (2013). Radiographic outcomes of volar locked plating for distal radius fractures. J Hand Surg Am.

[25] Raudasoja L, Vastamäki H, Raatikainen T (2018). The importance of radiological results in distal radius fracture operations: Functional outcome after long-term (6.5 years) follow-up. SAGE Open Med..

[26] Kim JK, Al-Dhafer B, Shin YH (2021). Effect of pre-treatment expectations on post-treatment expectation fulfillment or outcomes in patients with distal radius fracture. J Hand Ther.

[27] Castaing J (1964). Recent fractures of the lower extremity of the radius in adults. Rev Chir Orthop Reparatrice Appar Mot.

[28] Gartland JJJ, Werley CW (1951). Evaluation of healed Colles’ fractures. J Bone Jt Surg Am..

[29] Kazuki K, Kusunoki M, Yamada J (1993). Cineradiographic study of wrist motion after fracture of the distal radius. J Hand Surg Am.

[30] Synn AJ, Makhni EC, Makhni MC (2009). Distal radius fractures in older patients: is anatomic reduction necessary. Clin Orthop Relat Res.

[31] Thompson PW, Taylor J, Dawson A (2004). The annual incidence and seasonal variation of fractures of the distal radius in men and women over 25 years in Dorset. UK Injury.

[32] van Staa TP, Dennison EM, Leufkens HG (2001). Epidemiology of fractures in England and Wales. Bone.

[33] Wang WL, Ilyas AM (2020). Dorsal bridge plating versus external fixation for distal radius fractures. J Wrist Surg.

[34] Wentzensen A, Gebhard F, Grützner PA (2020). Spezielle Unfallchirurgie.

[35] Kong L, Kou N, Wang Y (2019). The necessity of restoration of radiologic parameters by closed reduction in elderly patients with distal radius fractures. Med Sci Monit.

[36] Yan B, Chen Y, Yin W (2019). Influence of distal radius fractures involving the intermediate column on forearm rotation. J Orthop Surg Res.

[37] Yang TY, Tsai YH, Shen SH (2014). Radiographic outcomes of percutaneous pinning for displaced extra-articular fractures of the distal radius: a time course study. Biomed Res Int.

[38] Jupiter (1997). Complex articular fractures of the distal radius: classification and management. J Am Acad Orthop Surg.

[39] Kibar B (2021). Combined palmar and dorsal plating of four-part distal radius fractures: our clinical and radiological results. Jt Dis Relat Surg.

[40] Kihara H, Palmer AK, Werner FW (1996). The effect of dorsally angulated distal radius fractures on distal radioulnar joint congruency and forearm rotation. J Hand Surg Am.

[41] Kim JK, Park MG, Shin SJ (2014). What is the minimum clinically important difference in grip strength. Clin Orthop Relat Res.

[42] Klum M, Wolf MB, Hahn P (2012). Normative data on wrist function. J Hand Surg Am.

[43] Lee YS, Wei TY, Cheng YC (2011). A comparative study of Colles’ fractures in patients between fifty and seventy years of age: percutaneous K-wiring versus volar locking plating. Int Orthop.

[44] Li Q, Ke C, Han S (2020). Nonoperative treatment versus volar locking plate fixation for elderly patients with distal radial fracture: a systematic review and meta-analysis. J Orthop Surg Res.

[45] MacDermid JC, Roth JH, Richards RS (2003). Pain and disability reported in the year following a distal radius fracture: a cohort study. BMC Musculoskelet Disord.

[46] Matschke S, Marent-Huber M, Audige L (2011). The surgical treatment of unstable distal radius fractures by angle stable implants: a multicenter prospective study. J Orthop Trauma.

[47] McQueen M, Caspers J (1988). Colles fracture: does the anatomical result affect the final function. J Bone Jt Surg Br.

[48] Moromizato K, Kimura R, Fukase H (2016). Whole-body patterns of the range of joint motion in young adults: masculine type and feminine type. J Physiol Anthropol.

[49] Myles PS, Troedel S, Boquest M (1999). The pain visual analog scale: is it linear or nonlinear?. Anesth Analg.

[50] Nishiwaki M, Tazaki K, Shimizu H (2011). Prospective study of distal radial fractures treated with an intramedullary nail. J Bone Jt Surg Am.

[51] Ruckenstuhl P, Bernhardt GA, Wolf M (2019). Influence of body mass index on health-related quality of life after surgical treatment of intra-articular distal radius fractures. A retrospective 7-year follow-up study. Hand Surg Rehabil..

[52] Soucie JM, Wang C, Forsyth A (2011). Range of motion measurements: reference values and a database for comparison studies. Haemophilia.

[53] Sriwatanakul K, Kelvie W, Lasagna L (1983). Studies with different types of visual analog scales for measurement of pain. Clin Pharmacol Ther.

